# Unintentional Suture Fistula Between the Proximal and Distal Esophagus in a Preterm Neonate with Type C Esophageal Atresia

**DOI:** 10.1055/a-2227-6252

**Published:** 2024-01-22

**Authors:** Julia E. Menso, Maud A. Reijntjes, Carlijn M. Mussies, Michiel P. van Wijk, Sander Zwaveling

**Affiliations:** 1 Department of Pediatric Surgery, Amsterdam UMC Location AMC, Amsterdam, North Holland, The Netherlands; 2 Department of Gastroenterology, Amsterdam UMC Location AMC, Amsterdam, North Holland, The Netherlands

**Keywords:** esophageal atresia, tracheoesophageal fistula, fistula formation

## Abstract

We present the case of a patient with gross type C esophageal atresia in a preterm neonate (gestational age of 31 weeks + 1 day) with a birth weight of 1,470 g. The fistula was released via a thoracotomy, but no primary anastomosis could be made, due to an unexpected long gap. The distal esophagus was closed and approximated to the blind pouch using traction sutures until an adjacent position was reached. A gastrostomy was created for enteral feeding. Although a second attempt to make an anastomosis was unsuccessful, the patient unexpectedly developed a suture fistula 6 weeks after the first procedure, enabling feeding via a nasogastric tube. Over time, six dilations were necessary. Full enteral feeding was achieved at the age of 6 months. Our case confirms sparse reports that deliberately creating a suture fistula may be a solution in esophageal atresia patients when an unexpected long gap prohibits a primary anastomosis.

## Introduction

In case of long gap esophageal atresia (EA; type A EA), we nowadays prefer to do a delayed repair (with initial gastrostomy) or a thoracoscopic Foker procedure. In our experience, this leads to better results than jejunal- or colonic replacement techniques, a gastric pullup procedure, or a cervical esophagostomy. In type C EA, usually a primary end-to-end esophago-esophageal anastomosis can be constructed. 1 2 3 In this type C EA case, however, we were unexpectedly not able to make a primary anastomosis. We had no previous experience with this situation and in this report, we describe our strategy and the course of events.

## Case Report

A male neonate (gestational age of 31 + 1) with a birth weight of 1,470 g was diagnosed with type C EA. VACTERL (vertebral, anal, cardiac, tracheal, esophageal, renal, and limb) screening showed no further anomalies. Three days postpartum, the patient underwent a surgery. Following sedation, a preoperative rigid tracheoscopy failed, because severe desaturation necessitated intubation. Via a thoracotomy, the tracheoesophageal fistula (TEF) was ligated and released from the trachea and the unusually short proximal pouch was mobilized. In spite of our efforts, it was impossible to directly make a primary anastomosis. The open end of the TEF was temporarily closed with interrupted sutures (Vicryl 4–0). Subsequently, by placing three traction sutures (Vicryl 4–0) through the tips of both ends, an attempt was made to close the gap. Once the proximal and distal parts touched, we decided to wait for 20 minutes for the tension to decrease. Upon inspection, we noted that one of the traction sutures had ruptured the wall of the proximal pouch. The defect was closed with interrupted sutures (Vicryl 4–0) and further attempts for a primary anastomosis were ceased. Both esophageal parts were left adjacent to each other. A gastrostomy was created for enteral feeding via a 6-Fr urine catheter.

**Fig. 1:**
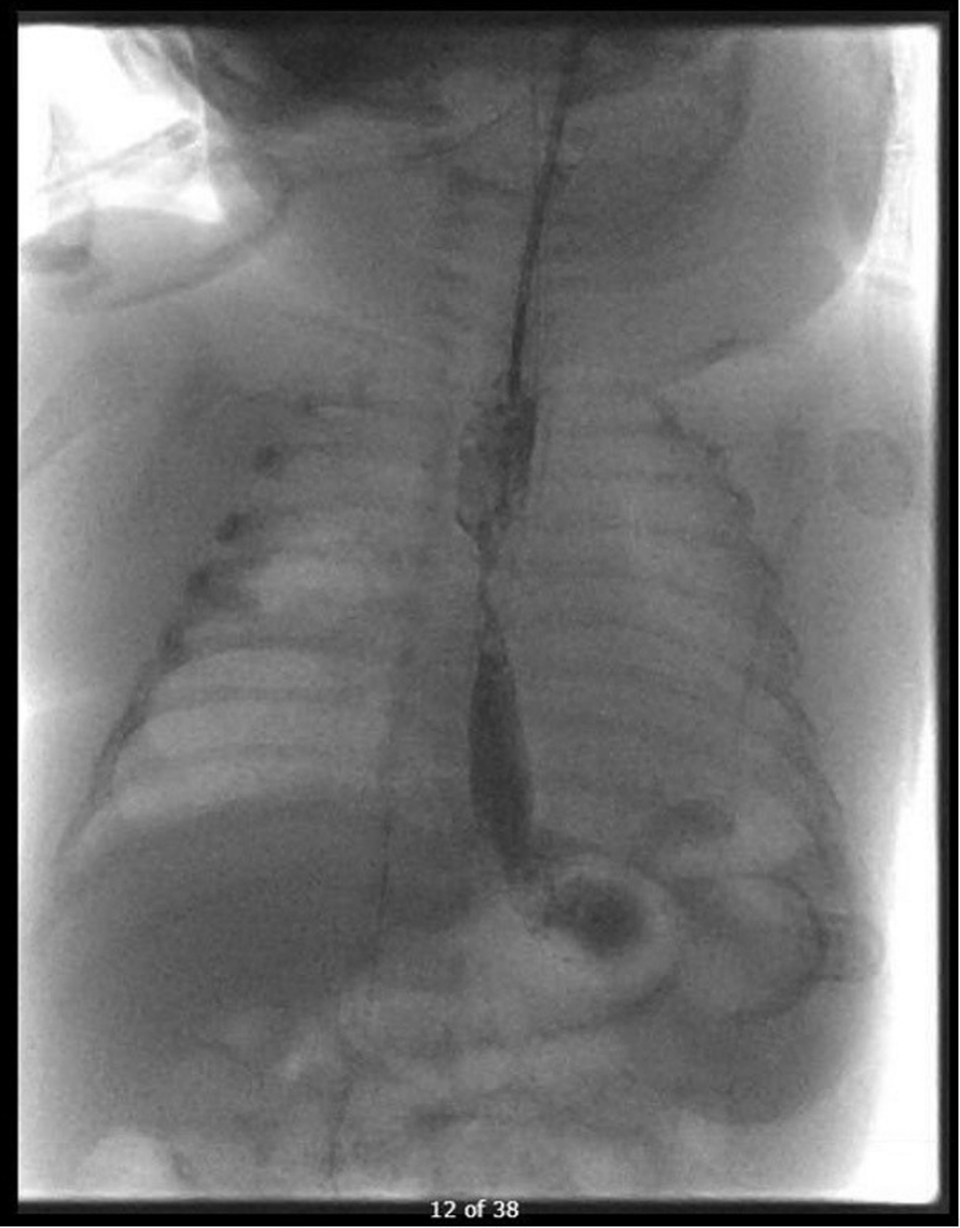
Contrast study after surgical approximation on which the suture fistula was first seen at 8 weeks of age.

**Fig. 2:**
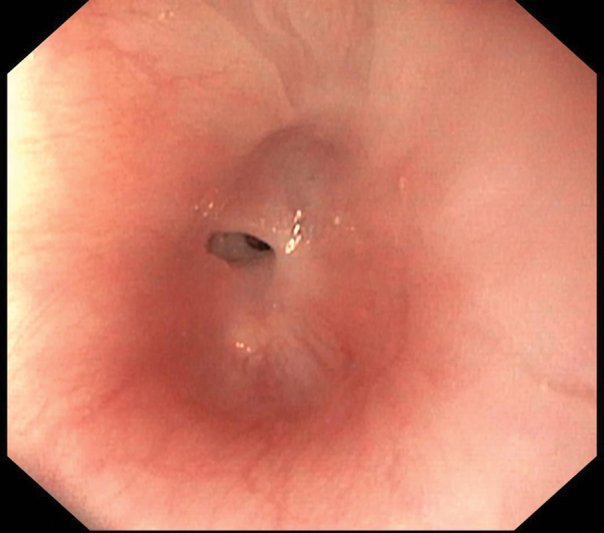
Screenshot during endoscopy demonstrating an open esophageal lumen before dilatation.

**Fig. 3:**
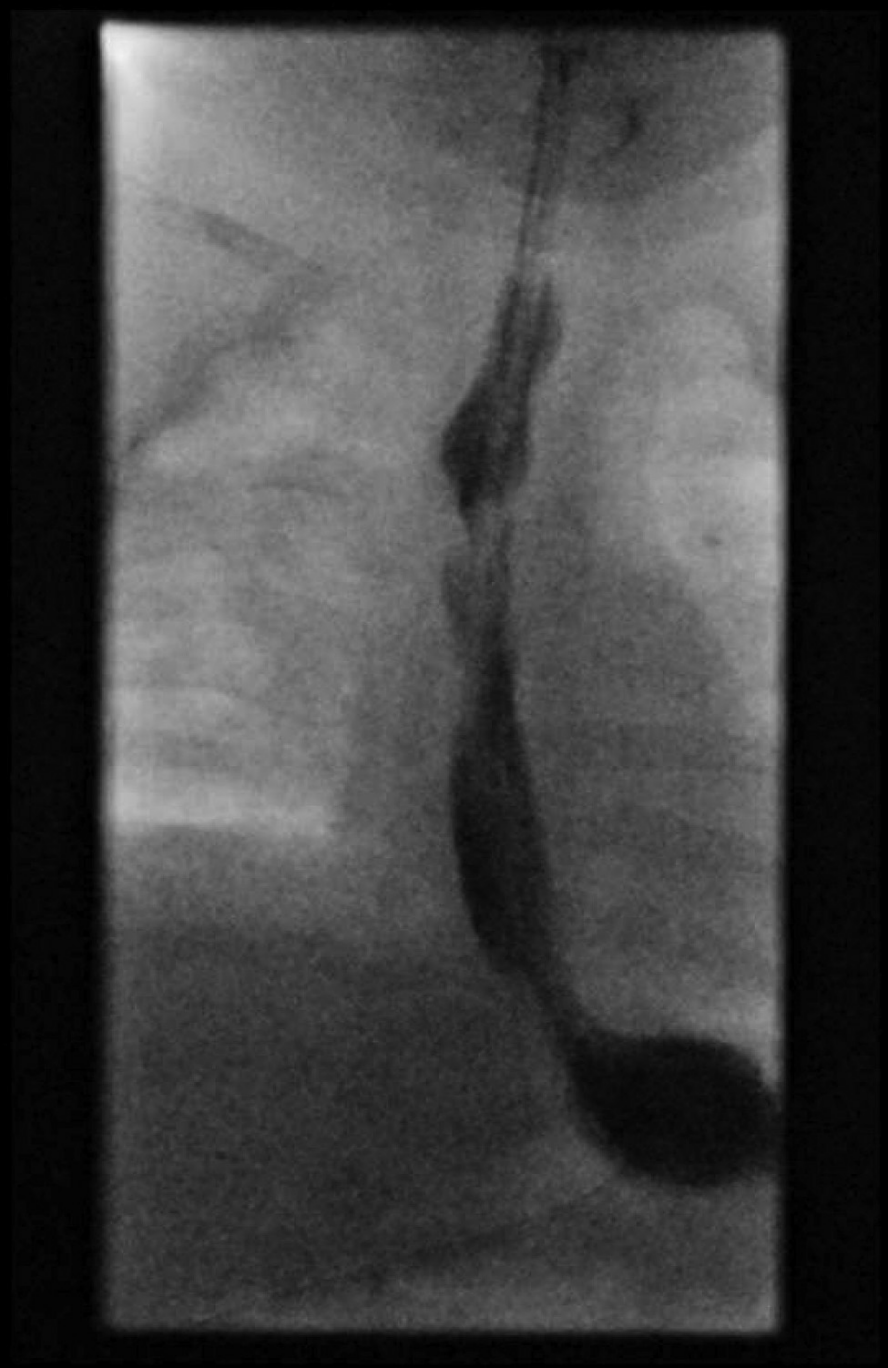
Contrast study showing no stenosis or prestenotic dilatation at 15 months of gestational age.

At the age of 6 weeks, a re-thoracotomy was attempted to make a delayed anastomosis. However, the thoracic cavity proved to be inaccessible due to adhesions and the procedure was stopped. This time, a preoperative rigid tracheoscopy was successful, showing a severe degree of tracheomalacia (95% decrease in lumen during inspiration) and a type 2 laryngeal cleft. Two weeks later, at the age of 8 weeks, milk was aspirated from the 10-Fr Replogle tube located in the proximal pouch. A contrast study with Omnipaque 240 mg I/mL demonstrated a short narrow caliber fistula between the proximal and distal esophagus ( Fig. 1 ). From the age of 11 weeks onward, six dilations (five under fluoroscopic and one under endoscopic guidance; Fig. 2 ) of the fistula were performed with Fogarty balloons ranging from 2.5 to 9 mm. Nasogastric tubes (initially 6 Fr, later 8 Fr) were left in place for enteral feeding. After the final dilation at the age of 5 months, the gastrostomy was closed to prevent leakage problems. In the meantime, an open aortopexy was performed at the age of 9 weeks. Although this seemed to be successful at first, the tracheomalacia recurred. Closure of the type 2 laryngeal cleft followed at the age of 16 weeks. A type 1 defect remained. Due to these associated respiratory problems, oral feeding was delayed and could not be started before the age of 6 months. By then, the patient had been admitted to a chronic care nursery and could finally be discharged at the age of 8 months weighing 6 kg. Although the infant still suffers from gastroesophageal reflux and esophageal dysmotility, no stenosis of the fistula has developed at 15 months of age as confirmed by contrast studies ( Fig. 3 ). The patient is now on full oral feeding.

## Discussion

In this patient with type C EA, initially we did not expect a long gap, and an open surgical approach was chosen based on the surgeon's preference. When we realized a primary anastomosis was not possible, we decided to approximate the proximal and distal esophagus and opt for a delayed anastomosis at a later stage. Alternatives such as esophageal replacement techniques or cervical esophagostomy may have been options, but these are associated with a high degree of morbidity. We presumed that in our patient over time the traction sutures unintentionally created a fistula, making an additional major procedure unnecessary. A review of the literature showed that the idea of deliberately creating a suture fistula in EA is not new. In 1974, Shafer and David described their suture fistula procedure, which involves approximation of the proximal and distal esophagus with a large central traction suture, resulting in fistula formation. 4 A recent report, describing three single-center cases since 1992, showed that this technique has been successfully applied in 24 patients since 1974. 5 A novel promising method to create a deliberate fistula in EA patients may be magnamosis, as recently reported by Conforti et al. 6 These strategies may serve as an escape when a primary anastomosis cannot be made.

## Conclusion

In conclusion, we describe the case of a long gap type C EA patient who developed an unintentional suture fistula between the proximal and distal esophagus after a failed primary closure attempt. No complications were noted and the fistula could be dilated to the size of a normal esophageal lumen (9 mm diameter). This prevented the child from having another major surgical procedure. Based on a literature search, deliberate formation of a fistula by sutures or magnamosis may be a solution in similar cases.
